# The Relationship between Change of Weight and Chronic Low Back Pain in Population over 50 Years of Age: A Nationwide Cross-Sectional Study

**DOI:** 10.3390/ijerph18083969

**Published:** 2021-04-09

**Authors:** Choung Ah Lee, Hae-Dong Jang, Ji Eun Moon, Sangsoo Han

**Affiliations:** 1 Department of Emergency Medicine, Dongtan Sacred Heart Hospital, Hallym University, 7 Keunjaebong-gil, Hwaseong-si 18450, Korea; cuccum@hanmail.net; 2 Department of Orthopaedic Surgery, Soonchunhyang University Bucheon Hospital, 170 Jomaru-ro, Bucheon 14584, Korea; khaki00@schmc.ac.kr; 3 Department of Biostatistics, Clinical Trial Center, Soonchunhyang University Bucheon Hospital, 170 Jomaru-ro, Bucheon 14584, Korea; moon6188@schmc.ac.kr; 4 Department of Emergency Medicine, Soonchunhyang University Bucheon Hospital, 170 Jomaru-ro, Bucheon 14584, Korea

**Keywords:** body weight changes, cross-sectional study, low back pain, odds ratio

## Abstract

Introduction: There is increasing evidence supporting an association between obesity and low back pain (LBP). However, the association between weight change and LBP in the general population is poorly understood. We investigated the relationship between weight change and LBP in a representative sample of the Korean general population from a nationwide survey. Methods: We analyzed data collected from the Korea National Health and Nutrition Examination Survey VI (2013–2015). Chronic LBP was defined as LBP lasting over 30 days in the last 3 months in the self-report health survey. Weight change was defined as the difference in weight from one year prior, and the amount of change was divided into no change, 3–6 kg, and ≥6 kg. Sampling weights were used to generate representative estimates for the general Korean population. Results: Overall, 6629 (12.0%) and 1848 (11.5%) participants were in the non-LBP and LBP groups, respectively. On multiple regression analysis, weight gain was significantly associated with LBP (adjusted odds ratio (OR) 1.29, *p* = 0.011), compared with no weight change. Weight gain of ≥6 kg was particularly closely associated with LBP (adjusted OR 1.42, *p* = 0.037), compared with no weight change. No association was found between LBP and weight loss. Conclusion: Weight gain is significantly associated with chronic LBP and, in particular, the greater the amount of weight gain, the stronger the association with an increased risk of chronic LBP. Clinicians should carefully monitor weight gain in LBP patients.

## 1. Introduction

Low back pain (LBP) is a common health problem that causes many inconveniences for those afflicted. LBP can lower the quality of life of an individual by causing discomfort in their daily life; it not only incurs direct healthcare costs but also poses an economic burden to society by reducing the available labor force [1,2]. The average annual incidence of LBP varies from 25% to 60%, and lifetime prevalence is reported in as much as 70% to 80% of the general population [3,4,5].

The etiology of LBP is multifactorial and complex, so it can rarely be identified; most LBP is referred to as nonspecific [6]. Multiple factors contribute to LBP, such as lifestyle factors (body mass index, smoking, drinking, and physical activity), socioeconomic factors (education level, occupation, and household income), and comorbidities [6]. Obesity is one of the factors associated with LBP, and, because the lumbar spine is one of the major joints that carry weight, LBP is frequently seen in obese patients [7]. One meta-analysis also reported that an individual being overweight is a strong risk factor for LBP [8]. Thus, LBP is believed to be closely related to weight, and especially to being overweight.

Most studies to date have shown a relationship between being overweight and low back pain, but few studies have been conducted on the relationship between weight change and low back pain [7,9,10]. Matsuda et al. [10] reported that excessive weight gain during pregnancy was a risk factor for persistent LBP. However, their study was conducted with a limited number of pregnant women. According to Dunlevy et al. [9], LBP decreased significantly in patients who lost more than 5% of their body weight through changes in lifestyle factors such as dietary guidance, psychology support, and support for increased physical activity. However, these results were not adjusted for other factors related to LBP, such as the effect of physical activity.

We aimed, therefore, to analyze the relationship between changes in weight (increase/decrease) and LBP in adults over 50 years old, correcting for many factors that are considered contributors to LBP, using nationwide survey data representing Korean adults. In addition, as a secondary outcome, we sought to understand the relationship between LBP and the magnitude of the weight increase or decrease.

## 2. Materials and Methods

### 2.1. Study Population

Data for this study were obtained from the Korea National Health and Nutrition Examination Survey (KNHANES) version VI, conducted from 2013 to 2015. The Korea Centers for Disease Control and Prevention (KCDC) have devised KNHANES to evaluate the health and nutritional status of Korean families every year since 1998; this is a national, clustered, multilevel, stratified, random sampling survey that samples the Korean population proportionally according to region, gender, and age group. Participants in KNHANES vary from year to year and are not continuously monitored. The survey includes approximately 10,000 independent samples from 192 primary sampling units (PSUs) every year; each PSU is selected using a sampling frame of all census blocks containing the Korean resident registration addresses. The questionnaire consists of three parts: health interviews with trained interviewers, medical examinations, and interviews with medical staff and laboratory technicians [11]. This study was conducted with the participants in KNHANES version VI, using the following exclusion criteria: under 50 years old; not having responded to the chronic LBP test questionnaire; not having responded to the weight change questionnaire.

### 2.2. Definition of Low Back Pain and Change of Weight

Chronic LBP was defined as a positive response (answering “yes”) to the following question: “Did you have low back pain for more than 30 days in the last 3 months?”

The change in weight was divided into three types according to the answer to the question: “Have there been any changes in your weight over the last year?” Answer options were as follows: no change; weight loss; weight gain. In the case of weight loss or gain, respondents were classified according to the changed weight as “3 kg to 6 kg” or “more than 6 kg”.

### 2.3. Description of Other Variables

We included lifestyle, socioeconomic factors, and comorbidities as covariates to control their effects as confounding factors. Body mass index (BMI) was calculated as weight divided by height squared, and was categorized as either underweight (<18.5 kg/m^2^), normal weight (18.5–24.9 kg/m^2^), and obese (≥25.0 kg/m^2^). Sleep duration was evaluated with the following question: how many hours do you generally sleep? Smoking status was categorized as current smoker, nonsmoker, or ex-smoker. Alcohol habit was classified according to the frequency of alcohol consumption: none, ≤1 drink/month, 2 drinks/month to 3 drinks/week, and ≥4 drinks/week. The educational level was divided into four groups based on the degree of graduation: ≤6 years (elementary school); 7–9 years (middle school); 10–12 years (high school); ≥13 years (university or college). Occupations were categorized into five groups: unemployed; office workers; sales and services; machine fitting and simple labor; agriculture, forestry, and fishery [12]. Household income level was categorized into four groups according to quartiles. Physical activity was defined as either mid-intensity physical activity for at least 2 h 30 min per week, high-intensity physical activity for >1 h 15 min per week, or a combination of middle- and high-intensity physical activity for a greater time period than stated above (1 min of high-intensity activity is equal to 2 min of mid-intensity activity) [13]. Medical history regarding major comorbidities such as hypertension, diabetes, dyslipidemia, stroke, myocardial infarction, angina, arthritis, asthma, depression, and malignancy such as lung, stomach, liver, colon, breast, or uterine cervical cancer, was checked in all participants.

### 2.4. Statistical Analysis

Student’s *t*-test was used to compare the continuous variables such as age, height, weight, BMI, and duration of sleep after checking normality using Shapiro–Wilk test, and chi-square test was used to compare categorical variables such as age group, sex, obesity, smoking status, alcohol consumption, education level, occupation, household income, physical activity, change of weight, amount of weight loss, amount of weight gain, and comorbidities. Multiple logistic regression analysis was performed to determine the relationship between chronic LBP and change of weight; odds ratio (OR) was calculated with 95% confidence intervals (CI). Multiple logistic regression was analyzed with three models to confirm the difference between the confounding variables caused by LBP. Model 1 was unadjusted; model 2 was adjusted by age and sex; model 3 was fully adjusted by age, sex, and other environmental factors including obesity, smoking, alcohol consumption, educational level, household income, occupation, physical activity, duration of sleep, and comorbidities. We also analyzed the relationship between chronic LBP and amount of weight loss or gain (no change, 3 kg to 6 kg, and ≥6 kg) using the multiple logistic model. Sampling weights were applied in the analyses so that the estimates were representative of the Korean population. Statistical significance was defined as *p* < 0.05. Statistical analyses were applied using IBM SPSS Statistics for Windows, version 26.0 (IBM Corp., Armonk, NY, USA).

## 3. Results

**Figure 1:**
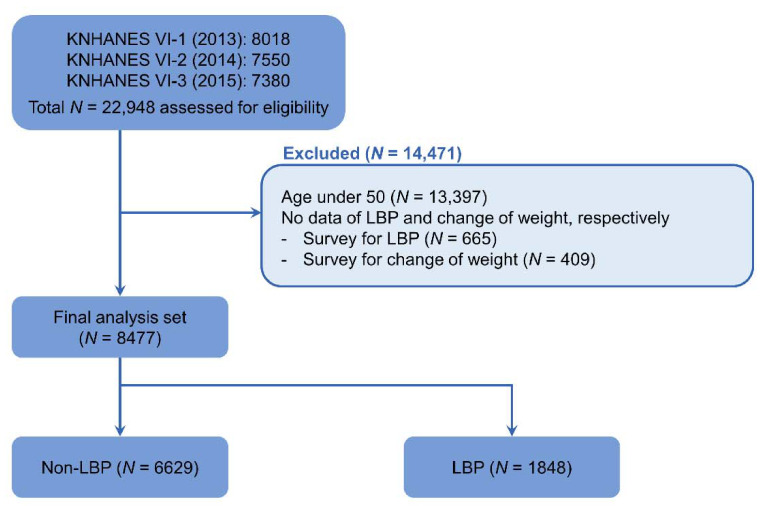
Flow chart of study subjects from the 2013 to 2015 Korea National Health and Nutrition Examination Surveys (KNHANES VI-1, VI-2, and VI-3). LBP, low back pain.

KNHANES IV-1 (2013), IV-2 (2014), and IV-3 (2015) examinations and health surveys were conducted with 8018, 7550, and 7380 individuals participating, respectively, for a total of 22,948 participants during the study period. According to the exclusion criteria, 13,397 people under the age of 50, 665 people without an LBP survey, and 409 people who did not complete the change of weight survey were excluded. This gave a final number of 8477 participants analyzed (Figure 1).

### 3.1. General Characteristics of Participants According to Low Back Pain

Of the total 8477 participants, 6629 were classed as non-LBP and 1848 as LBP. Among the weight change categories, weight loss was 14.4% in non-LBP and 16.8% in LBP. Weight gain was 13.5% among non-LBP and 17.2% for LBP. The degree of weight change was as follows: in the case of weight loss, a 3–6 kg decrease was reported for 14.2% of non-LBP and 15.3% of LBP, and ≥6 kg decrease was 3.4% in non-LBP and 5.0% on LBP. In the case of weight gain, non-LBP was 13.9% and LBP was 16.4% for 3–6 kg increase, and non-LBP was 2.8% and LBP was 4.3% for ≥6 kg increase. Age, sex, height, weight, BMI, obesity, duration of sleep, smoking status, alcohol consumption, education level, occupation, household income, physical activity, and comorbidities were significantly different between the two groups (Table 1).

### 3.2. Association between Change of Weight and Low Back Pain

**Figure 2:**
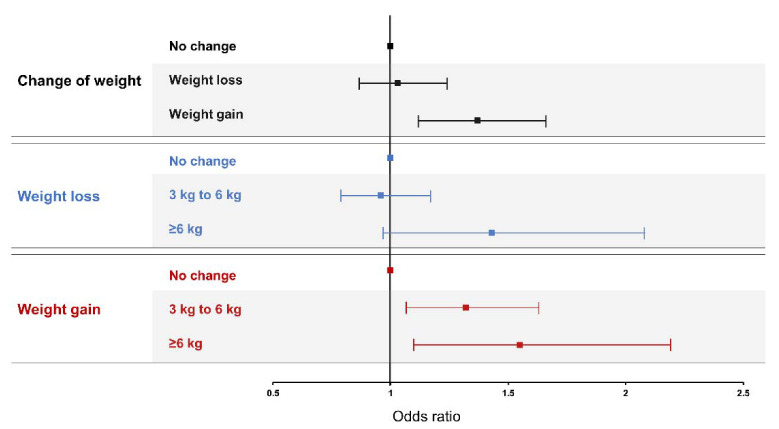
Association between change of weight and low back pain. Model was adjusted by age, sex, and other environmental factors such as obesity, smoking, alcohol consumption, educational level, household income, occupation, physical activity, duration of sleep, and comorbidities.

Table 2 shows estimated ORs for LBP from multiple regression analysis. Fully adjusted OR between weight gain and LBP was 1.29 (95% CI 1.06–1.57, *p* = 0.011). There was no correlation between weight loss and LBP in Model 3 (Figure 2).

For the relationship between LBP and the amount of change in body weight, the fully adjusted OR was 1.24 times higher (95% CI 1.01–1.53, *p* = 0.042) in the case of a 3–6 kg increase compared with the no change group. The fully adjusted OR of ≥6 kg increase group was 1.42 times higher (95% CI 1.02–1.98, *p* = 0.037) than that of the no change group. In the case of weight loss, there was no association with LBP, even according to the amount of change (Table 3, Figure 2).

## 4. Discussion

In this large, population-based cross-sectional study from the KNHANES database in South Korea, weight gain was found to be associated with higher risk of chronic LBP in adults over 50 years of age. The risk of chronic LBP was also associated with the amount of weight gain: in the case of a weight gain of 6 kg or more in the last year, the risk was 1.42 times higher than where there was no weight gain. On the other hand, weight loss did not show any association with chronic LBP.

Chronic LBP has a host of contributing factors [6]. First are lifestyle factors such as obesity, smoking, alcohol consumption, physical activity, and sleep duration. One meta-analysis study, for example, found that smokers had a higher prevalence of LBP than nonsmokers [14]. Alcohol consumption and physical inactivity have also been reported to be related to LBP [15,16,17], and a nationwide cross-sectional study stated that sleep duration, whether too short or too long, is associated with a higher risk of LBP [18]. Secondly, there are socioeconomic factors such as household income, educational level, and occupation. According to a cross-sectional study conducted in the United States, LBP was positively associated with education of less than high-school, and household income lower than USD 20,000 [19]. Third, the comorbidities can also be related. Mental disorders such as depression can be associated with chronic LBP. In a cross-sectional study using representative population sample in Germany, depression was reported as an independent predictor for disabling chronic LBP [20]. In our study, all possible factors that might contribute to LBP were adjusted for; these included age, sex, and environmental factors like obesity, smoking, alcohol consumption, educational level, household income, occupation, physical activity, duration of sleep, and comorbidities. In addition, we confirmed that the odds ratio (OR) for LBP decreased as more factors were adjusted in the three models, suggesting that LBP can indeed be mediated by these factors.

In this study, weight gain and the amount of weight gain showed correlation with chronic LBP. There are several possible explanations for the mechanism by which weight gain relates to LBP. First, as weight increases, a load is placed on the joint carrying the weight, and compression of the intervertebral disc may be induced due to axial loading on the lumbar spine, one of the major joints carrying the weight, and this may lead to injury [7]. Second, weight gain can cause spinal malalignment, especially lumbar lordosis, leading to LBP [21,22]. Third, the increase in adipose tissue as the body weight increases secretes cytokines such as tumor necrosis alpha and interleukin 6, which contribute to the development of pain via the alteration of neurophysiological properties of peripheral nociceptors and central neurons [23].

Previous studies have shown that losing weight is reported to help improve LBP [9,24]. In this study, however, weight loss or the amount of weight loss did not show any association with chronic LBP. This is likely because our study defined LBP as a positive reply to the question, “Have you had low back pain for more than 30 days in the last 3 months?” The research question was thus not helpful in determining whether weight loss improved the back pain, and additional studies will be needed to answer that question.

The major strength of this study is that it utilized a nationwide database to investigate the association between changes in body weight and LBP, using a large sample size representative of the Korean population. To the best of our knowledge, this is the first study to examine the relationship with chronic LBP according to changes of body weight and the amount of such change.

There are several limitations to this study. First, because this study is a retrospective cross-sectional design study, the causal relationship cannot be determined. Second, since the LBP was examined through only a simple questionnaire, the severity was not confirmed. Third, in KNHANES-VI, the questionnaire on LBP was conducted only with those participants over the age of 50. If younger people had been involved, the results might have been different. Fourth, since there is no information on seasonal, circannual rhythms and yoyo weight cycling in the KNHANES database, we could not consider such factors. Fifth, we were not able to stratify LBP according to severity, such as complaint only or disabling. To overcome these limitations, a large-scale, well-designed study is needed in the future.

## 5. Conclusions

In adults over 50 years of age, weight gain was significantly associated with a higher risk of chronic LBP. In particular, the more severe the weight gain, the more closely it was associated with the risk of chronic LBP. Therefore, in the treatment of LBP patients, clinicians may wish to prioritize education aimed at preventing weight gain.

## Figures and Tables

**Table 1:** General characteristics of the study population.

Variables	Non-LBP	LBP	*p*-Value
	(*N* = 6629)	(*N* = 1848)	
Age, year	63.18 ± 8.82	67.01 ± 9.02	<0.001
Age group, *N* (%)			<0.001
0–59	2662 (40.2)	477 (25.8)	
60–69	2220 (33.5)	540 (29.2)	
70–79	1407 (21.2)	654 (35.4)	
≥80	340 (5.1)	177 (9.6)	
Sex, *N* (%)			<0.001
Male	3134 (47.3)	475 (25.7)	
Female	3495 (52.7)	1373 (74.3)	
Height, cm	160.24 ± 8.73	155.84 ± 8.5	<0.001
Weight, kg	61.94 ± 10.33	59.44 ± 10.06	<0.001
BMI, kg/m^2^	24.06 ± 3.1	24.42 ± 3.36	<0.001
Obesity, *N* (%) *			0.0209
Underweight (<18.5)	169 (2.5)	47 (2.5)	
Normal (18.5–24.9)	4109 (62.0)	1081 (58.5)	
Obese (≥25)	2351 (5.5)	720 (39.0)	
Duration of sleep, h	6.64 ± 1.47	6.37 ± 1.68	<0.001
Smoking status, *N* (%)			<0.001
Non-/Ex-smoker	5598 (84.5)	1642 (88.9)	
Current smoker	1031 (15.5)	206 (11.1)	
Alcohol consumption, *N* (%)			<0.001
None	2475 (37.3)	921 (49.8)	
≤1 drink/mo	1665 (25.1)	465 (25.2)	
2 dirnks/mo to 3 drinks/wk	1915 (28.9)	353 (19.1)	
≥4 drinks/wk	574 (8.7)	109 (5.9)	<0.001
Education level, *N* (%) ^†^			
≤6 year	2432 (37.4)	1142 (61.8)	
7–9 year	1223 (18.1)	281 (14.9)	
10–12 year	1846 (28.0)	304 (16.1)	
≥13 year	1128 (16.5)	121 (7.2)	
Occupation, *N* (%)			
Unemployed	2996 (46.7)	1151 (62.5)	<0.001
Office work	751 (10.8)	86 (4.5)	
Sales and services	767 (11.1)	161 (8.7)	
Agriculture, forestry, and fishery	1257 (18.9)	240 (13.0)	
Machine fitting and simple labor	858 (12.5)	210 (11.3)	
Household income, *N* (%) ^‡^			<0.001
Low	1701 (25.7)	831 (45.0)	
Low–moderate	1790 (27.0)	457 (24.7)	
Moderate–high	1538 (23.2)	294 (15.9)	
High	1600 (24.1)	266 (14.4)	
Physical activity, *N* (%)	1925 (30.8)	391 (21.3)	<0.001
Change of weight, *N* (%)			<0.001
No change	4778 (72.1)	1219 (66.0)	
Weight loss	954 (14.4)	311 (16.8)	
Weight gain	897 (13.5)	318 (17.2)	
Amount of weight loss, *N* (%)			<0.001
No change	4778 (82.4)	1219 (79.7)	
3 to 6 kg	771 (14.2)	234 (15.3)	
≥6 kg	183 (3.4)	77 (5.0)	
Amount of weight gain, *N* (%)			<0.001
No change	4778 (88.9)	1219 (79.3)	
3 to 6 kg	745 (13.9)	252 (16.4)	
≥6 kg	152 (2.8)	66 (4.3)	
Comorbidities, *N* (%)			
Hypertension	2369 (35.7)	860 (46.5)	<0.001
Diabetes	886 (13.4)	361 (19.5)	<0.001
Dyslipidemia	1410 (21.3)	541 (29.3)	<0.001
Stroke	246 (3.7)	128 (6.9)	<0.001
Myocardial infarction	97 (1.5)	41 (2.2)	0.0304
Angina	187 (2.8)	105 (5.7)	<0.001
Arthritis	1156 (18.4)	788 (42.6)	<0.001
Asthma	180 (2.7)	128 (6.9)	<0.001
Depression	286 (4.3)	208 (11.3)	<0.001
Malignancy	194 (2.9)	55 (3.0)	0.973

Numeric parameters are expressed as mean ± standard deviation, and categorical parameters are expressed as counts and percentages in parentheses. BMI, body mass index; LBP, low back pain.* Body mass index was categorized into underweight (<18.5 kg/m^2^), normal (18.5–24.9 kg/m^2^), and obese (≥25.0 kg/m^2^).^†^ Educational level was divided into the following four groups: ≤6 years (elementary school), 7–9 years (middle school), 10–12 years (high school), and ≥13 years (college or university).^‡^ The household income level was measured at the level when compared with the standard amount for each age, and then was grouped into quartiles.

**Table 2:** Association between change of weight and low back pain using multiple logistic regression.

Group	Model 1			Model 2			Model 3		
	OR	95% CI	*p*-Value	OR	95% CI	*p*-Value	OR	95% CI	*p*-Value
Change of weight									
No change	1			1			1		
Weight loss	1.26	1.06–1.50	0.008	1.25	1.05–1.50	0.013	1.07	0.87–1.24	0.432
Weight gain	1.35	1.13–1.61	0.001	1.39	1.16–1.67	<0.001	1.29	1.06–1.57	0.011

OR, odds ratio; CI, confidence interval. Model 1 was an unadjusted odds ratio. Model 2 was adjusted by age and sex. Model 3 was fully adjusted by age, sex, and other environmental factors such as obesity, smoking, alcohol consumption, educational level, household income, occupation, physical activity, duration of sleep, and comorbidities.

**Table 3:** Association between the amount of weight change and low back pain using multiple logistic regression.

Group	Model 1			Model 2			Model 3		
	OR	95% CI	*p*-Value	OR	95% CI	*p*-Value	OR	95% CI	*p*-Value
Weight loss									
No change	1			1			1		
3 to 6 kg	1.06	0.88–1.27	0.534	1.10	0.90–1.34	0.362	0.99	0.81–1.12	0.937
≥6 kg	1.12	0.82–1.52	0.475	1.53	1.05–2.23	0.026	1.47	0.98–2.13	0.059
Weight gain									
No change	1			1			1		
3 to 6 kg	1.27	1.05–1.54	0.012	1.32	1.08–1.61	0.006	1.24	1.01–1.53	0.042
≥6 kg	1.73	1.24–2.42	0.001	1.75	1.24–2.47	0.002	1.42	1.02–1.98	0.037

OR, odds ratio; CI, confidence interval. Model 1 was an unadjusted odds ratio. Model 2 was adjusted by age and sex. Model 3 was fully adjusted by age, sex, and other environmental factors such as obesity, smoking, alcohol consumption, educational level, household income, occupation, physical activity, duration of sleep, and comorbidities.

## Data Availability

The data are available from the KCDC and Prevention database on the following webpage: https://knhanes.cdc.go.kr/knhanes/sub03/sub03_02_05.do (accessed on 3 March 2021). The data are available via this web page to anyone who meets the appropriate qualifications.
